# A tailored online safety and health intervention for women experiencing intimate partner violence: the iCAN Plan 4 Safety randomized controlled trial protocol

**DOI:** 10.1186/s12889-017-4143-9

**Published:** 2017-03-21

**Authors:** Marilyn Ford-Gilboe, Colleen Varcoe, Kelly Scott-Storey, Judith Wuest, James Case, Leanne M. Currie, Nancy Glass, Marilyn Hodgins, Harriet MacMillan, Nancy Perrin, C. Nadine Wathen

**Affiliations:** 1 Arthur Labatt Family School of Nursing, University of Western Ontario, FNB 2302, 1151 Richmond St., London, ON NBA 5C1 Canada; 2 School of Nursing, University of British Columbia, Vancouver, BC Canada; 3 Faculty of Nursing, University of New Brunswick, Fredericton, NB Canada; 4 School of Nursing, Johns Hopkins University, Baltimore, MD USA; 5 Departments of Psychiatry and Behavioural Neuroscience, and Pediatrics, Offord Center for Child Studies, McMaster University, Hamilton, ON Canada; 6 Faculty of Information and Media Studies, University of Western Ontario, London, ON Canada

**Keywords:** Intimate partner violence against women, Randomized controlled trial, Online interventions, e-health, Safety planning, Mental health, Technology, Mastery, Self-efficacy, Public health informatics, Computerized decision support

## Abstract

**Background:**

Intimate partner violence (IPV) threatens the safety and health of women worldwide. Safety planning is a widely recommended, evidence-based intervention for women experiencing IPV, yet fewer than 1 in 5 Canadian women access safety planning through domestic violence services. Rural, Indigenous, racialized, and immigrant women, those who prioritize their privacy, and/or women who have partners other than men, face unique safety risks and access barriers. Online IPV interventions tailored to the unique features of women’s lives, and to maximize choice and control, have potential to reduce access barriers, and improve fit and inclusiveness, maximizing effectiveness of these interventions for diverse groups.

**Methods/Design:**

In this double blind randomized controlled trial, 450 Canadian women who have experienced IPV in the previous 6 months will be randomized to either a tailored, interactive online safety and health intervention (i*CAN Plan 4 Safety*) or general online safety information (usual care). *iCAN* engages women in activities designed to increase their awareness of safety risks, reflect on their plans for their relationships and priorities, and create a personalize action plan of strategies and resources for addressing their safety and health concerns. Self-reported outcome measures will be collected at baseline and 3, 6, and 12 months post-baseline. Primary outcomes are depressive symptoms (Center for Epidemiological Studies Depression Scale, Revised) and PTSD Symptoms (PTSD Checklist, Civilian Version). Secondary outcomes include helpful safety actions, safety planning self-efficacy, mastery, and decisional conflict. In-depth qualitative interviews with approximately 60 women who have completed the trial and website utilization data will be used to explore women’s engagement with the intervention and processes of change.

**Discussion:**

This trial will contribute timely evidence about the effectiveness of online safety and health interventions appropriate for diverse life contexts. If effective*, iCAN* could be readily adopted by health and social services and/or accessed by women to work through options independently. This study will produce contextualized knowledge about how women engage with the intervention; its strengths and weaknesses; whether specific groups benefit more than others; and the processes explaining any positive outcomes. Such information is critical for effective scale up of any complex intervention.

**Trial registration:**

Clinicaltrials.gov ID NCT02258841 (Registered on Oct 2, 2014).

## Background

Intimate partner violence (IPV), a pattern of physical, sexual and/or emotional abuse in the context of coercive control [1], affects 1 in 3 Canadian women in their lifetimes [2] and often persists even after separation [3]. Over time, chronic stress and fear associated with experiencing IPV contributes to depression, anxiety, and Post-Traumatic Stress Disorder(PTSD) [4, 5]. In a meta-analysis of studies of female IPV victims, the mean prevalence of depression was estimated at 47.6%, and of PTSD at 63.8% (three- to five-fold and five-fold increases over general female population rates, respectively) [4].

Recognizing that a relationship is abusive and sorting out what to do about it is often a long, complex process during which women learn by trial and error about the risks to their physical and emotional safety and health, the resources and supports available to them, and the consequences of various strategies [6, 7]. Women’s capacity to address their safety is intertwined with their health. For example, women with poorer mental health are more likely to return to an abusive partner after initially separating [8]. To improve the mental health and quality of life of women experiencing IPV, gender- and context-specific, culturally safe interventions are urgently needed.

However, few interventions have been shown to improve the safety, health or quality of life of women experiencing IPV. Safety planning, including education to increase women’s awareness about IPV, and personalized support to identify options for reducing risks and support for accessing resources, is one of the most widely recommended IPV interventions [9]. There is evidence from systematic reviews that providing information and support to engage women in safety planning and accessing services that meet their needs reduces both physical and psychological violence [9, 10], but the effects on women’s mental health and well-being are unknown. Although violence-specific services are a key source of help with safety planning, fewer than 1 in 5 Canadian women who experience IPV access such services [11]. Rural, Aboriginal, racialized and immigrant women, women with partners other than men, and those who prioritize privacy (including more affluent women) face unique safety risks and barriers to accessing information and support for safety planning [12–15]. Effective, accessible, personalized interventions are needed to support women’s capacity to engage in actions to promote their safety and well-being, and potentially, to improve their sense of confidence, control and mental health.

Effective online health interventions have been developed in varied domains, including smoking cessation, depression, anxiety and sexual health [16–18]. Evidence shows that interaction, feedback, and tailoring are important features of these online interventions [17]. For women experiencing IPV, personalized, online interventions could improve access to information and support for safety planning and well-being, particularly for women who are reluctant or unable to access formal resources. In a recent pilot test of the first interactive, online safety decision aid for women experiencing IPV, called IRIS, Glass, Eden, Bloom and Perrin [19] found that women’s safety planning increased and decisional conflict decreased immediately after one use of this resource. The effectiveness of IRIS in improving the mental health of women exposed to IPV was recently tested in a randomized controlled trial in the United States [20], the results of which are pending. Two other variations of this online intervention (isafe and I-DECIDE) are currently being tested in randomized controlled trials in New Zealand [21] and Australia [22].

Drawing on these online interventions and our longstanding history of conducting research with women who have experienced IPV, we developed an evidence-based online safety and health intervention for diverse Canadian women experiencing IPV, called ‘*iCAN Plan 4 Safety’ (*hereafter referred to as *iCAN)*. In the development phase, we drew on insights from our completed research examining women’s lives and health after separation from abusive partner [23–27], including research to develop and test a complex health promotion intervention (called iHEAL) designed to support health and quality of life of women transitioning out of an abusive relationship [28, 29], and current research testing an adaptation of iHEAL for Indigenous women with histories of IPV [30]. In developing the intervention, we drew on these studies, our theoretical grounding and current literature to: a) broaden the concept of women’s safety, shifting beyond a focus on immediate physical risks to emphasize safety as an ongoing issue for women (including after separation), especially emotional safety, and a variety of issues that are closely linked to safety concerns, including women’s health and well-being over time; b) focus on developing a personalized (tailored) intervention that would be inclusive of and ‘fit’ with the uniqueness and complexity of Canadian women’s lives, particularly those facing the most substantial barriers to support; c) adopt an approach that recognizes and further builds women’s strengths and capacities, including their awareness of their own priorities and risks; knowledge of a wide range of options for addressing safety, health and life concerns; and confidence in their ability to try out these actions, while keeping control and choice in women’s hands. During the development phase, the online intervention was refined by iteratively integrating feedback about both content and usability until it was deemed acceptable by potential end-users. In this paper, we describe the trial protocol for testing the effectiveness of our online safety and health intervention (*iCAN*) among Canadian women.

### Objectives of the trial

The primary aim of this study is to test the effectiveness of *iCAN,* a tailored, online safety and health intervention, in improving the health and safety actions of adult women experiencing IPV. A group of women completing *iCAN* will be compared to a control group who receive general online safety information as a proxy for usual care. We hypothesize that, compared to the control group, women in the intervention group will: 1) have reduced symptoms of depression and PTSD (primary outcomes), and increase self-efficacy for safety planning, use of helpful safety actions, and mastery (secondary outcomes) at 6 and 12 months post-baseline; and 2) reduced decisional conflict immediately post-baseline (after completing the intervention one time) and at 6 and 12 months post-baseline. Additional aims are to: a) describe how women engage with the intervention, and their perceptions of its strengths and weaknesses; b) examine whether repeat engagement or dose are related to intervention effects; c) explore whether specific groups of women benefit in more or different ways from the intervention than others; and, d) examine the processes of change that explain how any positive outcomes are achieved.

## Methods

### Trial design

A two arm, double-blind randomized controlled trial is being conducted to test the effectiveness of *iCAN* in a diverse sample of Canadian women who are experiencing IPV. The protocol was developed using CONSORT e-health guidelines [31]. Outcomes will be measured pre-intervention (baseline) and 3, 6 and 12 months post-intervention; decisional conflict will also be measured immediately post-intervention (after a single completion of the intervention). Website utilization data are being collected to capture access to and use of the intervention and control conditions. Women’s experiences of engaging with the intervention and control condition will be explored after completion of the 12-month study visit using their ratings of potential benefits and harms and comments about their experiences (collected online) and via in-depth qualitative interviews conducted with a sub-sample of approximately 60 women. Ethics approval for this study was obtained in July 2014 from the Institutional Research Ethics Boards at the University of Western Ontario, University of British Columbia, and University of New Brunswick.

### Participant inclusion criteria

The target population for this trial is English speaking women aged 19 years or older, living in the provinces of Ontario, New Brunswick, or British Columbia who self-identify as experiencing IPV (physical, sexual or emotional abuse within the context of coercive control) by a current or former partner (boyfriend, girlfriend, husband, wife, or intimate partner) in the previous 6 months. Exposure to IPV will be determined by a positive response to at least one question on a modified 4-item version of the Abuse Assessment Screen, a standardized IPV screening tool that has been used in both research and clinical settings [25, 32, 33]. Specifically, women will be asked whether, in the previous 6 months, a partner or ex-partner ever: a) hit, kicked or otherwise physically hurt her; b) forced her to have sexual activities against her will; c) did things to make her feel afraid of them; or d) did things to try and intimidate her or to control her thoughts, feelings or actions. Eligible participants must have access to a safe computer (to access the study website), safe email address (to which study-related information will be sent), and a safe mailing address (e.g., that of a friend or family member) to which the participation fee can be sent. Women not meeting all of these criteria will be excluded from participation.

### Recruitment

Women will be recruited to the trial using advertisements posted in a variety of online spaces (e.g., classified ads, health or violence websites, online discussion forums), supplemented by more traditional approaches to advertising in community settings (e.g., libraries), or through organizations, associations or service agencies accessed by women. Women seeking more information about the study will be directed to the study website (www.icanplan4safety.ca) or asked to contact a Research Assistant (RA) by email or a toll-free telephone number. This approach is similar to that utilized by the IRIS trial, and mirrors the varied ways that women might discover and access the online intervention outside of a research context.

### Number of participants required

A sample of 450 women (225 per group) will be recruited for this trial based on the statistical power needed to both test the impact of the tailored online intervention on primary outcomes using a general estimating equations (GEE) approach, and, subsequently, to examine mechanisms by which the intervention exerts its effects on these outcomes using Structural Equation Modeling (SEM). We based the power analysis on two key outcomes: decreased symptoms of depression, and of PTSD. Baseline means and standard deviations from the IRIS study [34], a similar trial in the USA, were used in the power analysis. In estimating the sample size, we varied the percent change over time in the intervention group relative to the control group from 15% to 20% which corresponds to moderate effects sizes, 0.29 and 0.52, found in previous studies of IPV interventions. The power analysis was based on repeated measures ANOVA since specific estimates of variance needed to calculate sample size for GEE are not available. However, in the GEE we will be able to retain all cases in the analysis, including those with missing data. With an alpha of .05, 15% greater change in the intervention group, relative to the control group, a sample size of 400 participants provides .73 power for depression and .92 power for PTSD. With a 20% greater change in the intervention, power is .94 for depression. We assume that attrition will be approximately 10%, based on the retention rate in the recently completed US trial (<10% at 12 month follow up) [35] and our recent success in retaining 81% of a cohort of women who had experienced IPV over a 4- year period. Attrition of 10% at 12 months would result in a final sample of 405 women, adequate for all analyses.

### Trial process

Women who are interested in taking part in this study will contact a RA by telephone. The RA will review the study purpose with each woman, assess her eligibility using a Recruitment and Eligibility Form, and invite all eligible women to participate. Initially, the RA will obtain informed verbal consent by reading the letter of information to the woman and answering questions she may have; as noted below, women will provide written consent to participate when they login to the website. Each woman who consents will have her name, safe email address and preferred contact information, province of residence, whether she has children or not, and how she learned about the study recorded on the Eligibility and Recruitment Form by the RA. The RA will discuss safety concerns with the woman and develop a plan for safe contact with the study. The RA will also enter this information into an electronic tracking database, housed on a secure server at The University of Western Ontario. The woman will then be asked to create a security question and answer for the purpose of identification in the case of a lost password.

Entry of the woman’s safe email address into the tracking database will initiate her enrollment into the study. At this point, the system will generate a unique study ID, randomly assign the woman to either the intervention or control group, and send out an automated ‘welcome’ email to the woman’s safe email address containing a link to the letter of information and consent, and a corresponding URL, username and password. The women will be instructed to use these credentials to login to the website. The first time the woman logs into the website, she will be asked to re-affirm her consent to participate by selecting 3 radio buttons indicating that: a) she has read the letter of information, b) has had all questions about the study answered, and, c) agrees to participate. These questions must be answered affirmatively in order to proceed.

At baseline, the woman will be encouraged to login and complete the baseline measures and intervention activities on the same day she is randomized and receives her study website password. The woman will be allowed to complete the baseline study visit in more than one sitting; completed responses will be automatically saved so that the woman can continue where she left off when she logs in at other times. However, she will have 6 weeks from the time of enrollment in which to complete the baseline measures and accrue to the trial. Automated and manual emails will be sent at regular intervals over the 6-week period until the woman either completes baseline measures or the time expires. Should the time expire, an email message will be sent to inform the woman that she is no longer able to complete the tool. Based on the IRIS trial and our pilot study, baseline study measures are expected to take about 30 min to complete. The additional time required to complete intervention activities is 30-60 min for women in the intervention arm and 20-30 min for those in the control arm.

For 3, 6 and 12-month follow-up, automated reminder emails will be sent to the woman’s safe email address notifying her that she is due to complete another session. The woman’s username and password, along with the study URL will be included in the email notification. When the woman returns to the website, the study measures will be slightly different for the 3, 6 and 12 month assessments. At these follow-up visits, women in the intervention group will be invited to complete the intervention activities again if they wish, and encouraged to consider this option if their situations have changed. Once enrolled, women will be able to visit their assigned website between scheduled study visits to review their responses. Women in the intervention arm will be able to update some activities, such that the online tool can be used as an ongoing resource that changes and evolves to fit with their changing life contexts (see Intervention arm for details).

Website utilization data for both study arms will be collected automatically to: 1) track the number of times the site is accessed within scheduled follow-up windows and between windows; 2) the time spent on a session and within specific sections; 3) item completion rates; and, 4) materials accessed and skipped. This information will be used to describe the how women engage with the intervention and control websites as well as inform us on dosing.

### Randomization and blinding

On enrollment, participants will be automatically computer randomized using a stratified blocked randomized scheme based on whether she has children or not and province of residence using information gathered during recruitment that has been entered into the electronic tracking data base by a RA. Once participants are randomly allocated to either the intervention or control group, they will be automatically sent a link to the appropriate website. Women will not be informed of their group assignment, although they may guess which online intervention they are using based on the extent to which they receive personalized (versus general) information and suggestions. Members of the research team, with the exception of the programmer (JC) and statistician (NP), will also be blind to the group allocation of participants until 12-month data have been collected so that research staff, who may be contacted by women for technology or other support, are not in a position to provide preferential treatment to women based on group assignment.

### Online Interventions

The online interventions that will be delivered to women in both arms are organized into the same sections and focus on: 1) questions about women’s background characteristics; 2) women’s plans for their relationship with their abusive partner, their priorities and current safety risks; 3) an action plan containing safety planning information and resources, and 4) a structured debriefing. The content of each section by study arm is summarized in Table 1, with details of each provided in the text that follows.

**Table 1:** Comparison of content of intervention and control arms

Section	Study arm		Study arms differ
	Tailored intervention	Usual care control	
Background questions	Questions about women’s demographic characteristics and living situation	Questions about women’s demographic characteristics and living situation	No
My decisions, priorities, risks	• Plan for Relationship • Priorities Exercise with personalized feedback • Danger Assessment with personalized feedback	• Plan for Relationship • General Information about Priorities • General Information about risk of IPV	No Yes Yes
My action plan	Strategies organized in 8 categories with associated resources • Recommended based on her responses to questions and activities; woman can further customize	Emergency safety planning and child safety strategies and resources • General information not personalized to the woman’s situation; no opportunity to modify	Yes
Debriefing	Standardized information about symptoms of a stress reaction and strategies for managing	Standardized information about symptoms of a stress reaction and strategies for managing	No

#### Intervention arm (tailored, online safety and health intervention)

Women will initially respond to questions about their personal context (such as whether they have children, are employed, were born in Canada). In the next section, “*My Decision, My Priorities, My Risks*”, women will complete a series of questions and interactive activities. First, women will be asked to reflect on their relationship with their abusive partner and to report on their current plan from 5 options (i.e., stay with partner, separate, remain separated, return to partner, unsure). Second, women will be invited to complete an interactive priority setting exercise in which they will rate the relative importance of 5 factors (my feelings for my partner, my health and well-being, having resources, my concern for safety, and my children’s well-being) in making decisions about their unsafe relationship. Women will be shown pairs of priorities positioned at opposite ends of a horizontal line and then slide a toggle bar toward the priority that is more important to them. Weights for each priority will be computed mathematically and real time feedback provided in the form of a graph, along with examples of safety strategies in the action plan that fit with the top priority identified. Third, women will complete the Danger Assessment (DA), or DA-Revised (for women with partners other than men), a validated risk assessment [36, 37]. Using the DA, women will be asked to mark the type and timing of episodes of physical and/or sexual violence from their partner in the past 12 months on a calendar, and then respond to 19 questions assessing risk factors for serious or lethal violence. A standardized weighted score (range 0-38), calculated using these responses, will then be provided to the women on a graph with information about the corresponding level of risk, ranging from variable danger to extreme danger, and a brief personal message about their level of danger and suggested actions.

Next, in “*My Action Plan”*, women will create a tailored Action Plan for themselves by selecting from 46 strategies organized in 8 different groups (Emergency Plan; Domestic Violence Information and Services, Security Measures, Basic Resources, Health, Support from Family, Friends and Community, Child Safety and Legal Strategies). Each strategy conveys detailed information about actions women could take to address their safety and health concerns. To engage diverse groups of women, these strategies are intentionally written in a conversational tone, use a coaching approach that includes ‘tips’ for accessing services (e.g., how specific services work and how to access them more quickly) or using a strategy (e.g., a guided video to demonstrate grounding exercises), and comments about conditions that might alter the strategies (e.g., If you live in a rural community…..”). Linked resources include contact and other information for services, and relevant websites and videos. When women begin this exercise, some strategies will be marked as recommended for each woman based on her unique responses to previously completed questions and activities (e.g., whether parenting children, plan for the relationship, DA score). Women will be instructed to select each strategy to open and read it, and to further personalize their action plan by selecting additional strategies important to them, and/or removing (de-selecting) those which they do not want to keep in their plan. Encouraging customization by the woman is an approach that reinforces her control in creating the plan. Women will be encouraged to save, print or access the Action Plan online at any time if safe to do so, and to visit the website to update their Action Plans should their circumstances change.

As the final step in the intervention, women will be provided with information about the signs of a stress reaction, advised that this is a normal reaction that may occur following a study visit, and provided with suggestions for managing this type of reaction, if it occurs. This is part of a standardized debriefing protocol we have used successfully in other studies [38] and an important step in promoting women’s emotional safety when engaging with the study.

#### Control arm (General Online Risk and Safety Planning Information)

Women in the control arm will complete the same background questions and question about their plans for the relationship as those in the intervention arm. However, in Section 2, which focusses on decisions, priorities and risks, brief general information will be provided about the importance of considering priorities when making important decisions along with risk factors for IPV, but women will not complete the interactive Priorities Exercise or the DA, or be provided with individualized feedback. Next, women will be provided with an Action Plan containing emergency safety strategies (1 of 8 categories of strategies provided to the intervention group), including safety of children, and some general information about online and community resources that is not tailored to the woman’s situation. The type of information included will be consistent with what women would find online if they did their own search. In this sense, it constitutes a proxy for usual care. As with the intervention group, women will be able to save or print their plan for later use, or revisit the website at any time during the 12-month period of the study. After the final study visit has been completed, women in the control group will be offered an opportunity to complete the tailored, online intervention and provided with a clickable link to access it if they wish.

### Retention

We will use a retention protocol based on proven techniques for tracking women in longitudinal IPV studies [25]. Retention strategies will include: a) asking women for names and contact information for up to 6 people (family, friends, employers, service providers) who would know of their whereabouts and could be used as alternate safe contacts, along with their consent to contact people to release this information to us, b) contacting participants mid-way between the 4 study visits (at 1.5, 4.5, and 9 months post baseline) to confirm contact information and any anticipated changes to this; c) providing a small incentive for completing each of the 4 study visits, with the value increasing over time (i.e., $20, $30, $40 and $50); and, d) after each email or telephone contact, providing the woman with the name of the local RA, the study phone number and email, and the date of the next study visit. Efforts will be made by the RAs to use friendly, warm communication strategies to establish rapport with participants. It is hoped that by using telephone-based enrollment, women will feel more comfortable with the research team, and be more likely to ask for support if needed, improving retention.

### Safety and security

The physical and emotional safety of women who participate in the *iCAN* Plan 4 Safety trial is a primary concern. We will adopt many safety procedures used in the IRIS trial [19] with success (i.e., that trial reported no adverse events). All research staff will be trained in the use of these safety procedures. We expect the most significant safety risks faced by women in the trial will be from an abusive partner who becomes angry if they discover that she is participating in this research. A number of strategies will be used to prevent risk of detection by abusive partners and to ensure that participation is private and confidential. Information will be provided to all participants regarding the safe (private) use of computers and the Internet. As necessary, the RA will help the woman brainstorm a safe location to assess a computer or tablet (e.g., family, friend, public library, community agency, work, etc.). Women will be encouraged to not use a computer or tablet where the partner could potentially witness her participating in the study or access her Internet history. The RA will also offer training to the women about how to safely use the Internet. For example, the RA will provide information about deleting browser history and send this information to women via email if requested; this information will also be posted on the home page of the study website. When women load the *iCAN* website, a pop up dialogue box appears with information and instructions for opening the site in private mode (Incognito mode). The website is also equipped with a ‘quick escape’ bar that when clicked, leads to two actions: 1) provides immediate exit from the website and brings the user to the Google homepage; 2) pushes the tab running *iCAN* to the weather network webpage. All manual and automated emails sent to participants are addressed with the subject header “Women’s Health Study” to further reduce risk of detection.

Given the potential for questions about sensitive topics to trigger women with experiences of violence and trauma, and their experiences of stigma and judgment by others, women’s emotional safety was also considered in developing both the online surveys and the interventions to limit any potential emotional harms of participating. The language and content of the surveys and intervention was carefully drafted and reviewed to ensure inclusiveness and applicability to women from various backgrounds so that women could see themselves in the study, and to avoid phrasing that could be interpreted as blaming or stigmatizing. To increase women’s sense of comfort, we attempted to use an informal, conversational tone, included messages that acknowledged when questions or activities might create distress for women, and encouraged women to take a break if needed or otherwise care for themselves. As previously noted, we added a structured debriefing at the end of each study visit to remind the woman that she might experience a stress reaction after the session, that this is a normal response and to provide options for managing it.

Risk of suicidality is heightened in women with histories of IPV [39]. Although we do not anticipate that study participation will increase this risk, we adapted a protocol developed for the IRIS study for use in both study arms. Safety programming has been integrated into the online interventions to: a) identify women who are at risk of suicide using their responses to standardized questions for depressed mood and suicide attempts, b) advise women directly if they are at-risk of suicide and provide options for managing the situation, including a follow up contact with an RA; and; c) alert RAs by email when a woman at-risk wishes to be contacted. Follow-up contact with women will be guided by a standard protocol, based on current best practices in suicide management. All research assistants will complete the Applied Suicide Intervention Skills Training (ASIST). The principal investigators at each site, who are Registered Nurses, will be available to consult with and support RAs, if needed.

An independent Data Safety Monitoring Committee (DSMC) will meet every 6 months to review reports of potential harms (e.g., exposure to abuse, risk of suicide) and adherence to safety study protocols prepared by our statistician. The committee will recommend investigations and/or follow up actions about any safety concerns which they identify to the Co-PIs (MFG, CV, KSS, JW).

### Outcomes

The primary outcomes are:

- *Depressive Symptoms* measured by the Center for Epidemiologic Studies Depression Scale, Revised (CESD-R) [40]. The CESD-R is a 20-item self-report measure of symptoms reflective of the DSM-IV criteria for depression. Women rate how often they have experienced each symptom in the past week using 5 options that range from ‘not at all or less than 1 day’ to ‘nearly every day for 2 weeks’. Total and subscale scores are computed by summing responses to applicable items. The revised CESD correlates highly with the original scale, and demonstrates good to excellent face and construct validity, as well as excellent internal consistency (α = .90-.96) [40, 41].
- *PTSD Symptomology*, measured on the PTSD checklist, Civilian Version (PCL-C) [42, 43]. The PCL-C is a 17 item self-report measure designed for use in community samples to assess the probability of meeting DSM-IV diagnostic criteria for PTSD. The PCL-C asks about symptoms in relation to generic stressful experiences; women rate how bothered they are by each symptom during the past month using a 5 point Likert-type scale, with a range of 1 (not at all) to 5 (extremely). Total summation score range from 17-85, with higher scores indicative of greater symptomatology. The PCL-C has demonstrated validity and excellent internal consistency reliability (.94 for the total scale and .82 to .94 for subscales) [44].

The secondary outcomes are:

- *Decisional Conflict*, measured on an adapted 13 item version of the low literacy Decisional Conflict Scale (DCS) [45]. The DCS assesses the extent to which women understand the advantages and disadvantages of safety planning options and their values related to these decisions [46]. The DCS discriminates between people who make decisions and those who delay making decisions [47]. Women self-report on a 3 point Likert-type scale as to which option they prefer, ‘yes’ (0), ‘unsure’ (2), or ‘no’ (4). Scoring is summative, with higher scores representing higher levels of decision conflict and anxiety [45]. The low literacy version of the DCS has shown good test-retest reliability (.75) and internal consistency (α = .72) [47].
- *Use of Safety Actions*, measured on a 22 item self-report scale, adapted from several sources, including the Safety Behavior Checklist [48], Intimate Partner Violence Strategies Index [49] and our previous longitudinal research [50]. Women are asked to indicate whether they have used a variety of safety behavior strategies/actions (yes/no) within the past 12 months and, if used, how helpful this strategy was it in dealing with the violence (on a 5-point scale ranging from ‘not at all helpful’ to ‘very helpful’).
- *Mastery*, measured on Pearlin’s 7-item Mastery Scale. The Mastery Scale is a self-report measure that taps into perceptions of personal control over one’s life. Women respond to how much they agree (1 = strongly agree) or disagree (7 = strongly disagree) with each item. Scores are computed by summing responses to all items, where higher scores indicating greater perceived mastery. The Mastery Scale has demonstrated good internal consistency (α = .75 -.78) and has been widely used in a variety of populations providing evidence of strong face validity [51–53].
- *Self-efficacy for Safety Planning*, measured on 2 visual analogue scales (VAS), developed for this study. Women are asked to rate their confidence in making a safety plan for themselves on a 100 mm horizontal line, with anchors of ‘not at all confident’ and ‘completely confident.” Women with children will be asked to complete a second VAS to rate their confidence in making a safety plan for their children. VAS scores are recorded by the website as the distance in mm from the left anchor (0) to the location of the mark on the line. Scores range from 0 to 100, with greater scores represent greater self-efficacy for safety planning.

Other outcomes:

- *Level of Coercive Control*, measured on the Women’s Experiences with Battering (WEB) Scale [54]. The WEB is a 10-item scale designed to measure the intensity of experiences of psychological vulnerability from IPV and the impact of coercive control [54–56]. For each item, woman are asked to respond on a 6-point Likert scale, ranging from 1 (Strongly agree) to 6 (Strongly disagree). Total summation scores range from 10 to 60; lower scores represent less abusive behavior and loss of power and control. The WEB has demonstrated high internal consistency (α = .99) and construct validity [54].
- *Severity of Intimate Partner Violence*, measured on the 30-item Composite Abuse Scale (CAS), asks women to rate the frequency of experiencing specific abusive acts in the previous 12 months on a 6-point scale ranging from ‘never’ (0) to ‘daily’ (5). Using cut-off scores, women’s responses are categorized as ‘positive’ or ‘negative’ for exposure to 4 types of IPV: physical abuse, emotional abuse, harassment and severe combined abuse. A total summative score can be derived, with higher scores indicative of greater severity of abuse [57]. The CAS has demonstrated good internal consistency [58] and evidence of content, construct, criterion, and factorial validity [59]. In this study, the 3 sexual abuse items were modified to make them more consistent with current theory and measurement approaches in the field [60].
- *Social Support*, measured using a 5-item modified scale from the Medical Outcomes Study Social Support Survey (MOS-SSS). The shortened MOS-SSS assesses the perceived availability of emotional, informational, and instrumental support. For each item, the availability of support is rated on a 5-point Likert-type scale, ranging from 1 (none of the time) to 5 (all of the time). Total summative scores are computed, with higher scores suggestive of greater perceived support. The 5-item version has demonstrated good internal consistency (α = .87) and validity [61].

### Data collection

Collection of outcome data will be integrated into both the intervention and control group websites and will be collected at baseline (prior to completion of the intervention) and 3-, 6-, and 12-months post-intervention. At each study visit, participants in both groups will complete self-report measures of mental health, decisional conflict, use of safety actions, self-efficacy for safety planning, social support, mastery, IPV severity and coercive control. Demographic information will be collected at baseline and updated at subsequent waves, if needed. Website utilization data for both study arms will be collected automatically to track the number of times the site is accessed, time spent, and materials accessed and skipped. This information will be used to describe how women engage with the intervention and control websites (including completion rates of specific sections). Feedback on the helpfulness of the online intervention will also be collected at the end of each study session. Women will be asked to complete a brief exit survey immediately following the 12-months study visit to gather their ratings of perceived comfort, harms and benefits of the research using questions adapted from other studies, and any additional comments they wish to provide.

### Statistical analysis

#### Examination of outcomes by study arm

The effectiveness of the intervention will be assessed by comparing the intervention and control groups on changes in outcome measures, between the baseline and the 3, 6, and 12 month post-baseline assessments. We will test the hypothesis that, at 3, 6 and 12 months post-baseline, the intervention group will have improved mental health (our primary outcomes), in comparison to the control group, using intent-to-treat principles with generalized estimating equations (GEE). The same approach will be used for testing secondary outcomes which hypothesize decreased decisional conflict, increased safety seeking behavior, mastery and self-efficacy. Separate analyses will be conducted for each outcome. The parameter of interest is the group (intervention vs. control) by time interaction, which if significant means that change over time differs for intervention and control groups. Significant findings will be graphed to determine the nature of the effect. Potential confounding variables for which intervention and control groups are significantly different at baseline and variables that are related to missingness will be entered in the model. These analyses will establish overall effect sizes, for specific outcomes, for the intervention.

#### Analysis of differential intervention effects

To test for differential effects of the intervention for specific subgroups of women, we will either enter a grouping variable (e.g., rural/urban) and its three-way interaction with intervention group and time, or, if subgroups are reasonably large, we will analyze differences in intervention effects using multi-group analysis techniques. These analyses are not fully powered; therefore, we will interpret differences in effect sizes across the specific subgroups rather than rely on statistical significance. Subgroups to be examined include geographic location (rural/urban), partner status (separated from partner or not), continuing exposure to abuse (yes/no), mothering (yes/no), Indigenous identification (yes/no).

#### Testing the mechanisms that explain the interventions effects

To better understand how the intervention achieves its effects, we will employ structural equation modeling (SEM) techniques for longitudinal data to test a theoretical model specifying the relationships between change in the intervention, a set of mediating variables (decisional conflict, safety strategies, support, mastery, abuse severity) and women’s mental health. Latent variables will be developed for each construct from existing manifest variables. Goodness of fit between the model and the data will be assessed. The repeated measures character of the data will be integrated in the model by specifying latent growth curve trajectories for the outcome and some of the mediating variables.

### Process evaluation

In-depth qualitative interviews will be conducted with a sub-sample of approximately 60 women after they have completed the trial to explore how they engaged with the intervention; their perceptions of intervention strengths and weaknesses, risks and benefits, and helpfulness; and processes of change that may explain intervention effects. At the end of the 12-month study visit, all participants (both groups) will be asked to indicate their interest in participating in a qualitative interview about their experiences taking part in the study, and to provide permission for a RA to contact them using their safe email address. Maximum variation sampling will be used to identify and recruit a sample of women with varied sociodemographic backgrounds (e.g., age, whether they are mothers, geographic location) and histories with their partners (e.g., abuse history, partner gender, living with partner versus separated) from those who consent to be contacted. A RA will contact these women to provide information about the interview process, seek separate informed consent and to arrange a time for the interview. Interviews will be conducted by an investigator or trained RA by telephone or Skype using existing study protocols for safe contact. A structured interview guide will be used to explore how women engaged with the online interventions and how information or resources were used; positive and negative aspects and impacts; and how the context of women’s lives shaped their intervention use and impacts. Interviews will be audio-recorded and transcribed verbatim and analyzed using content analysis techniques.

The process evaluation will also draw on data collected in the 12-month exit survey offered to all women, administrative (use) data collected by the online system (both groups), and report of helpfulness of the online interventions in supporting women’s decision making (collected at baseline and 3, 6, and 12-month study visits using the Preparation for Decision-Making Scale previously described) in order to fully develop, explain, and contextualize the findings.

## Discussion

This trial builds on and extends research on online safety interventions for women experiencing IPV in three key ways. First, the intervention tested in this study integrates increased attention to both health and emotional safety – both during completion of the online intervention and in the strategies offered to women as part of the intervention. Second, building on lessons learned from IRIS, we have streamlined data that will be collected from the control group women (e.g., no completion of the Danger Assessment), in an effort to reduce any measurement effects. Finally, we will be able to test for effects in a range of specific contexts, including varied geographies, women who have and have not separated from their abusive partners and who are/are not experiencing ongoing violence. Therefore, study findings will contribute timely evidence about the effectiveness of online safety and health interventions appropriate for diverse life contexts. Moreover, this study will produce contextualized knowledge about how women engage with the intervention; its strengths and weaknesses; whether specific groups benefit more than others; and the processes that explain any positive outcomes. If effective, this information is critical for successful scale up of complex interventions, such as *iCAN*, into health and social services and/or for public access for women who prefer to work through the intervention independently.

### Trial status

Active follow up.

## Abbreviations

IPV: Intimate partner violence
iCAN: iCAN Plan 4 Safety
RA: Research assistant
PTSD: Post-traumatic stress disorder
DA: Danger Assessment
